# Treatment effectiveness and preference in patients with schizophrenia switched from oral standard of care atypical antipsychotics to aripiprazole once-monthly: a brief report over a six-month period

**DOI:** 10.3389/fpsyt.2025.1680394

**Published:** 2025-12-03

**Authors:** Gianluca Mancusi, Andrea Miuli, Giulia Giovannetti, Ornella Di Marco, Daniele Di Battista, Teresa Di Crosta, Mauro Pettorruso, Giovanni Martinotti, Marco Alessandrini

**Affiliations:** 1 Department of Neuroscience, Imaging and Clinical Sciences, “G. d’Annunzio” University of Chieti, Chieti, Italy; 2 Department of Mental Health, Azienda Sanitaria Locale (ASL) Lanciano-Vasto-Chieti, Chieti, Italy; 3 Institute for Advanced Biomedical Technologies (ITAB), G D’Annunzio University of Chieti-Pescara, Chieti, Italy

**Keywords:** schizophrenia, aripiprazole, long acting, switch, side effect

## Abstract

**Background:**

Schizophrenia is a chronic and disabling psychiatric disorder characterized by poor treatment adherence, often leading to relapse and functional decline. Long-acting injectable antipsychotics (LAIs), such as aripiprazole once-monthly (AOM), offer a potential solution by improving adherence and reducing relapse rates. This study evaluates the clinical effectiveness, safety, tolerability, and patient satisfaction in individuals with schizophrenia who transitioned from oral standard-of-care (SOC) atypical antipsychotics to AOM over a six-month period.

**Methods:**

An open-label, single-arm, mirror-image study design was employed. Forty adult patients diagnosed with schizophrenia and previously stabilized on oral antipsychotics for at least two months were enrolled. Following a retrospective assessment of a six-month oral treatment period, patients were prospectively followed for six months after switching to AOM. Clinical assessments included the Investigator’s Assessment Questionnaire (IAQ), Clinical Global Impression-Severity (CGI-S) scale, and the Treatment Satisfaction Questionnaire for Medication (TSQM 1.4). Adverse effects were monitored via self-report and clinician-rated tools.

**Results:**

Twenty-five patients completed at least the first follow-up visit. A statistically significant improvement in IAQ scores was observed over time, indicating enhanced global clinical effectiveness (p=0.010 at six months). CGI-S scores also significantly decreased, reflecting reduced illness severity (p<0.001 at six months). Patient satisfaction, measured by TSQM, increased significantly at three and six months (p<0.001). AOM was well-tolerated, with only minor, non-significant changes in weight gain, extrapyramidal symptoms, and prolactin levels. Notably, negative symptom scores showed a modest but statistically significant improvement (p=0.032). Only five patients (18%) reported adverse events, mostly with moderate impact, and no treatment discontinuation was recorded.

**Conclusions:**

Transitioning to AOM from oral antipsychotics in schizophrenia patients was associated with significant improvements in clinical outcomes, symptom severity, and treatment satisfaction, with a favorable safety and tolerability profile. These findings support the use of AOM in early-stage schizophrenia and highlight its potential to enhance long-term adherence and functional recovery. Further long-term studies are warranted to assess the durability of these benefits and their impact on relapse prevention and quality of life.

## Introduction

Schizophrenia is one of the leading causes of global health burden being a chronic disorder with an early onset, characterized by significant disability and progressive functional decline, leading to severe consequences for patients’ lives (1, 2). Over the past century, the understanding of schizophrenia has evolved, with changes in its definitions and boundaries aimed at improving research, diagnosis and treatment, thereby fostering a better understanding of the disorder and its variants (3). Early intervention is crucial for treating schizophrenia patients to minimize disabilities and improve chances of recovering premorbid abilities (4, 5). Pharmacological treatment with antipsychotic drugs is essential in managing schizophrenia. However, successful outcomes largely depend on patient adherence, which is often suboptimal in this patient population (6). Given this scenario, long-acting injectable antipsychotics (LAIs) have been introduced to enhance adherence, consequently reducing relapse and hospitalization risks while improving patient functioning (7, 8).

Among the LAIs, aripiprazole once-monthly (AOM) is an atypical antipsychotic administered intramuscularly, well-known for its efficacy in treating schizophrenia and its ability to reduce the risk of relapse (9–12). Besides improving adherence to therapy through its once a month intramuscular administration, AOM significantly enhances the quality of life for patients diagnosed/living with schizophrenia (13–15). The observed beneficial effects of aripiprazole are due to its pharmacodynamic properties. For further clarification, aripiprazole is an antipsychotic drug acting as a partial agonist at D_2_ dopamine and 5-HT1_a_ serotonin receptors and as an antagonist at 5-HT2_a_ serotonin receptors resulting as a dopamine-serotonin system stabilizer (16).

In several studies, oral aripiprazole therapy, primarily administered during the initial phase of schizophrenia, has been compared to AOM in terms of efficacy and adherence, with AOM demonstrating more satisfactory outcomes and lower hospitalization rates (10, 17–20). Furthermore, patient preference in the choice of therapy type should also be taken into consideration (17). Therefore, it is crucial to compare the two modes of administration to assess both adherence levels and patient preferences, especially during the early stages of the disease.

The primary objective of this study is to evaluate the efficacy of AOM versus oral atypical antipsychotics standard of care (SOC) hypothesizing that AOM therapy would demonstrate a favorable profile in terms of efficacy, safety, and tolerability compared with SOC treatment. Furthermore, it will examine patient treatment preference, the severity of illness both before and after therapy transition, and the occurrence of any adverse events.

## Methods

### Participants

At the time of recruitment, all participants had been receiving oral aripiprazole or other oral antipsychotic therapy for at least six months and had maintained a stable clinical condition for at least two months prior to initiating aripiprazole once-monthly (AOM) treatment, with a disease duration of less than five years. The subjects were recruited from the mental health department of Azienda Sanitaria Locale (ASL) Lanciano-Vasto-Chieti, Italy, through referrals from their attending psychiatrists from January 2021 to April 2024. Patients with diagnoses of mood disorders, delirium, dementia, cognitive disorders, or significant neurological and medical conditions were excluded. Also, those who could not comply with study procedures were not selected. The sample included patients with a history of illness ranging from six months to a maximum of five years. Medical and psychiatric comorbidities were exclusion criteria; therefore, recruited patients did not present any relevant organic or systemic condition. One participant reported alcohol use disorder. Eligible subjects underwent clinical and psychometric assessments to evaluate the efficacy, safety, and tolerability of the therapy using the Investigator’s Assessment Questionnaire (IAQ) scale. Furthermore, to evaluate the disease state and satisfaction with the mode of therapy administration, subjects were assessed during the study phases using the Clinical Global Impression Severity (CGI-S) scale and the Italian version of the Treatment Satisfaction Questionnaire for Medication (TSQM 1.4). Additionally, adverse effects related to the therapy were assessed. The study was approved by the local ethics committee (protocol no. 2728 November 2020), the institutional review boards, and the national regulatory authorities, in accordance with local regulations. All participants were able to provide written informed consent in accordance with the Declaration of Helsinki.

### Study design

This was an open-label, single arm, single-center, non-interventional study that adopted a mirror-image methodology to assess the effectiveness of AOM in patients with schizophrenia. It involved retrospective treatment with oral SOC atypical antipsychotics for six months, followed by prospective treatment with AOM for an additional six months. As this was an observational study, clinical stabilization with oral aripiprazole at 10–30 mg for a minimum of two months was required prior to transitioning to AOM. During this phase, patients underwent an initial evaluation and were selected based on eligibility criteria. Informed consent was obtained during this visit, and a retrospective assessment was conducted to gather clinical and psychiatric information. Additionally, scales such as CGI-S and TSQM 1.4 were administered to evaluate patient status and treatment satisfaction, respectively, while adverse effects were also assessed. Upon achieving clinical stabilization, on the recommendation of the referring psychiatrists, subjects switched to AOM at a dosage of 400 mg via intramuscular injection in the gluteal or deltoid muscle, which could be subsequently reduced to 300 mg for better tolerability if needed. Concurrently, they received oral aripiprazole (10–20 mg) for the first 14 days of intramuscular therapy, according to a standardized cross-titration protocol aimed at ensuring therapeutic overlap and minimizing potential side effects. Subsequently, subjects were reassessed for adverse effects and underwent psychometric evaluations (IAQ, CGI-S, and TSQM 1.4) after three months of AOM therapy and the end of the study (6 months) (**Table 1**). Time points are described in detail in the Data Analysis section.

**Table 1:** Investigator’s Assessment Questionnaire (IAQ) test mean values.

IAQ - Descriptive statistics			
	Mean	Std. dev.	N
IAQ - T0	20.16	4.58	25
IAQ - T1	19.08	7.45	25
IAQ - T2	17.40	5.70	25

### Assessment tools

The questionnaires used in this study were the IAQ, the Clinical Global Impressions-Severity scale and Italian version of Treatment Satisfaction Questionnaire for Medication version 1.4.

The IAQ is a newly developed 10-item instrument designed to assess the relative effectiveness (encompassing efficacy, safety, and tolerability) of antipsychotic medications in patients with schizophrenia or schizoaffective disorder. The total IAQ score exhibits significant correlations with time to treatment discontinuation (21) and higher values of this scale, showing a better insight, could represent an index of global clinical improvement.

The Clinical Global Impressions-Severity (CGI-S) scale is a single item test performed by the clinician that assesses global impression of severity of illness ranging from 1 to 7: a score of 1 indicates the patient is normal and not at all ill, 2 represents borderline illness, 3 is mildly ill, 4 is moderately ill, 5 is markedly ill, 6 is severely ill, and 7 indicates the patient is among the most extremely ill (22).

**Figure 1:**
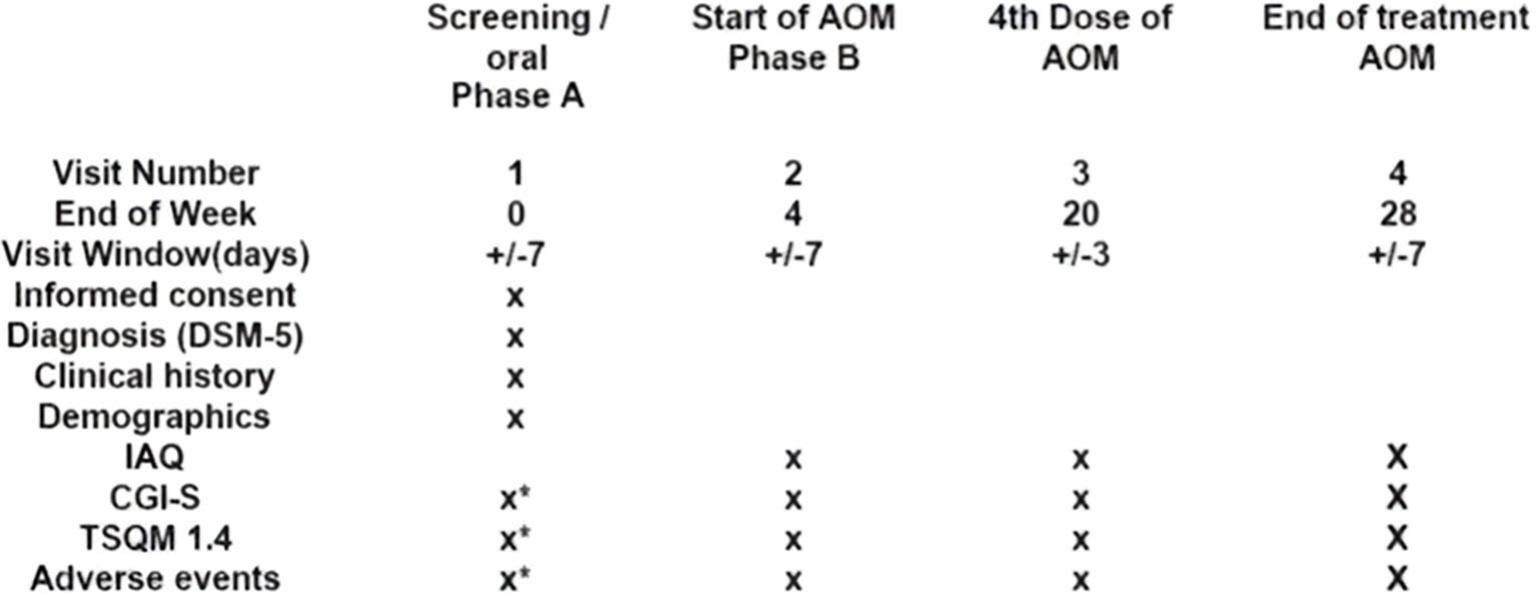
Study visits and procedures.

The Italian version of the Treatment Satisfaction Questionnaire for Medication (TSQM Version 1.4) is a patient-reported outcome tool used for self-assessment of treatment effectiveness, side effects, convenience, and overall satisfaction. This reliable and valid instrument comprises 14 items (23) (**Figure 1**).

### Data management

All enrolled patients were assigned a unique and progressive identification code (ID). Datasets were anonymized by removing all direct identifiers (e.g., name, address, telephone numbers) as well as indirect identifiers and any other information that could lead to deductive disclosure of participants’ identities. The computer files were password-protected and accessible only to authorized team members. These files were shared with other involved institutions via a secure server. Hard copies, such as interview notes, questionnaires, and psychometric scales, were securely stored in a locked cabinet accessible only to authorized research team members. As part of the informed consent process, participants were informed about the handling of their data, specifically: a) how the data would be stored; b) who would have access to the data; and c) the duration for which the data would be retained.

### Data analysis

Statistical analysis was performed using statistical packages, namely SPSS 19. Continuous variables have been presented as mean and standard deviations, categorical variables as number and percentage.

#### Primary outcome

A three time [baseline (T0), three months (T1), six month (T2)] repeated measure of Analysis of variance (RM-ANOVA) was performed, entering the IAQ total score as the dependent variable. Statistical significance threshold was put at p<0.05.

#### Secondary outcomes

A three time (T0, T1, T2) RM-ANOVA was performed on CGI-S score and TSQM 1.4 score, in order to identify any pre-post difference in terms of symptoms severity and patient’s satisfaction.

#### Adjunctive analyses

Number and type of adverse events in pre-post periods was recorded and compared using a chi-squared statistic.

## Results

Forty patients aged 18 to 65 years, both male and female, with a diagnosis of schizophrenia defined by the International Classification of Diseases 9 diagnostic criteria (ICD -9) were enrolled in the study. Among these, thirteen subjects withdrew their participation before the three-month follow-up (T1), and two withdrew before the six-month follow-up (T2). Statistical analysis was conducted on subjects who completed at least the first follow-up (N = 25). The analyzed sample consisted of 59.2% male individuals (N = 16) with a mean age of 40.4. The dosage of oral aripiprazole prescribed prior to AOM ranged from 10 mg to 30 mg (mean dosage: 14.09 mg). At the end of the observation period, two subjects reduced their dosage of AOM to 300 mg for tolerability reasons, while the rest of subjects received a monthly dosage of 400 mg.

**Figure 2:**
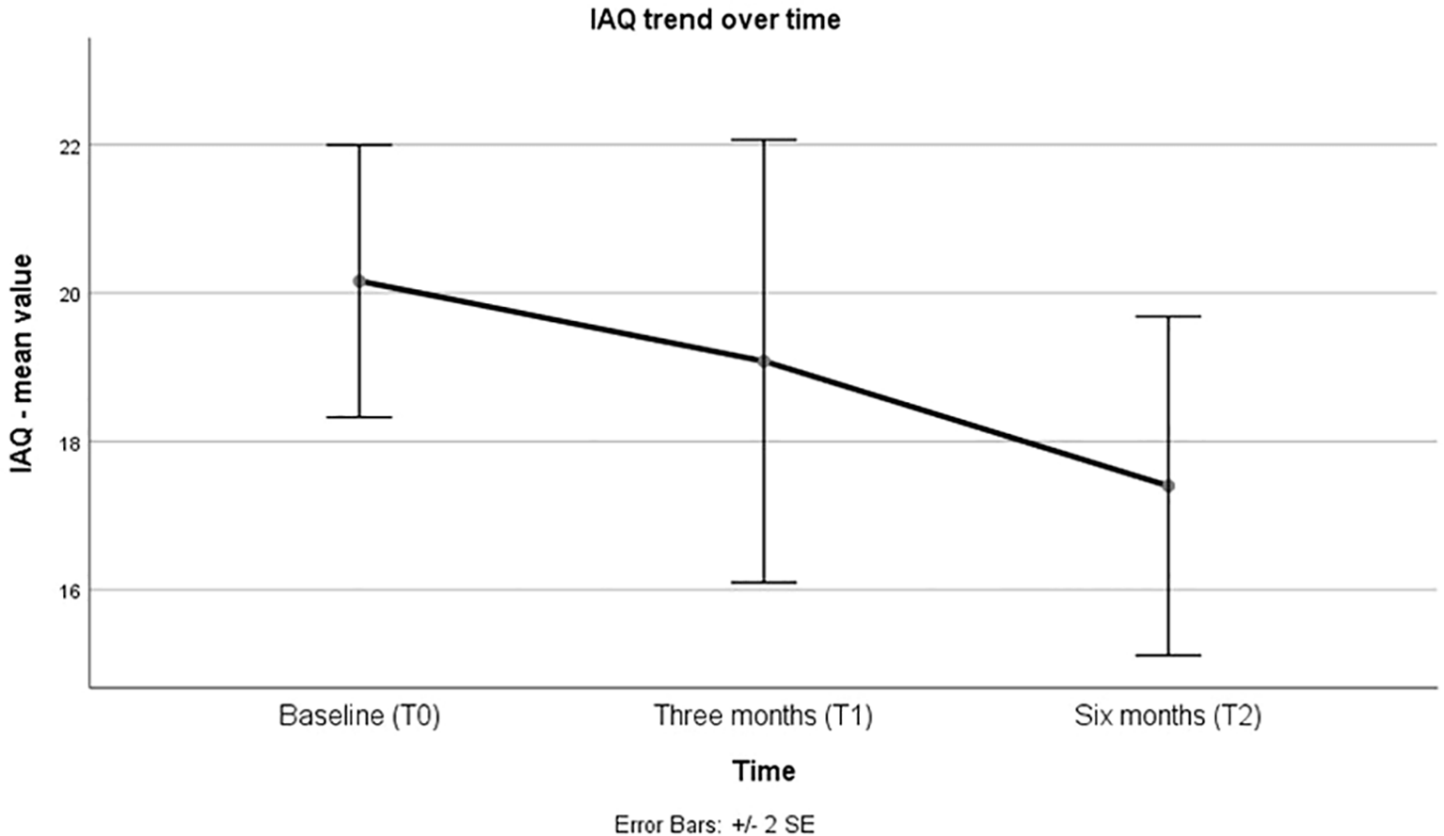
Variation of mean IAQ value over observation time.

The scores on the IAQ scale showed a progressive reduction at the first and second observations compared to baseline, representing a clinical improvement (**Table 1**; **Figure 2**). This difference was found to be significant in the RM-ANOVA test between T0 and T2 (**Table 2**). These results survive the Bonferroni adjustment.

**Table 2:** Repeated measurement ANOVA for IAQ test.

IAQ (T0 - T1 - T2) - RM-ANOVA						
Time		Difference of the mean	Std. error	Sign.^b^	95% CI	
					Inferior limit	Superior limit
T0	T1	1.08	0.98	0.846	-1.44	3.60
	T2	2.76^*^	0.84	0.010	0.58	4.95
T1	T0	-1.08	0.98	0.846	-3.60	1.44
	T2	1.68^*^	0.53	0.013	0.31	3.04
T2	T0	-2.76^*^	0.84	0.010	-4.93	-0.58
	T1	-1.68^*^	0.53	0.013	-3.04	-0.31

Based on the estimated marginal means.

^*^The difference of the mean is significant for p< 0.05.

^b^Adjustment for multiple comparisons: Bonferroni.

**Figure 3:**
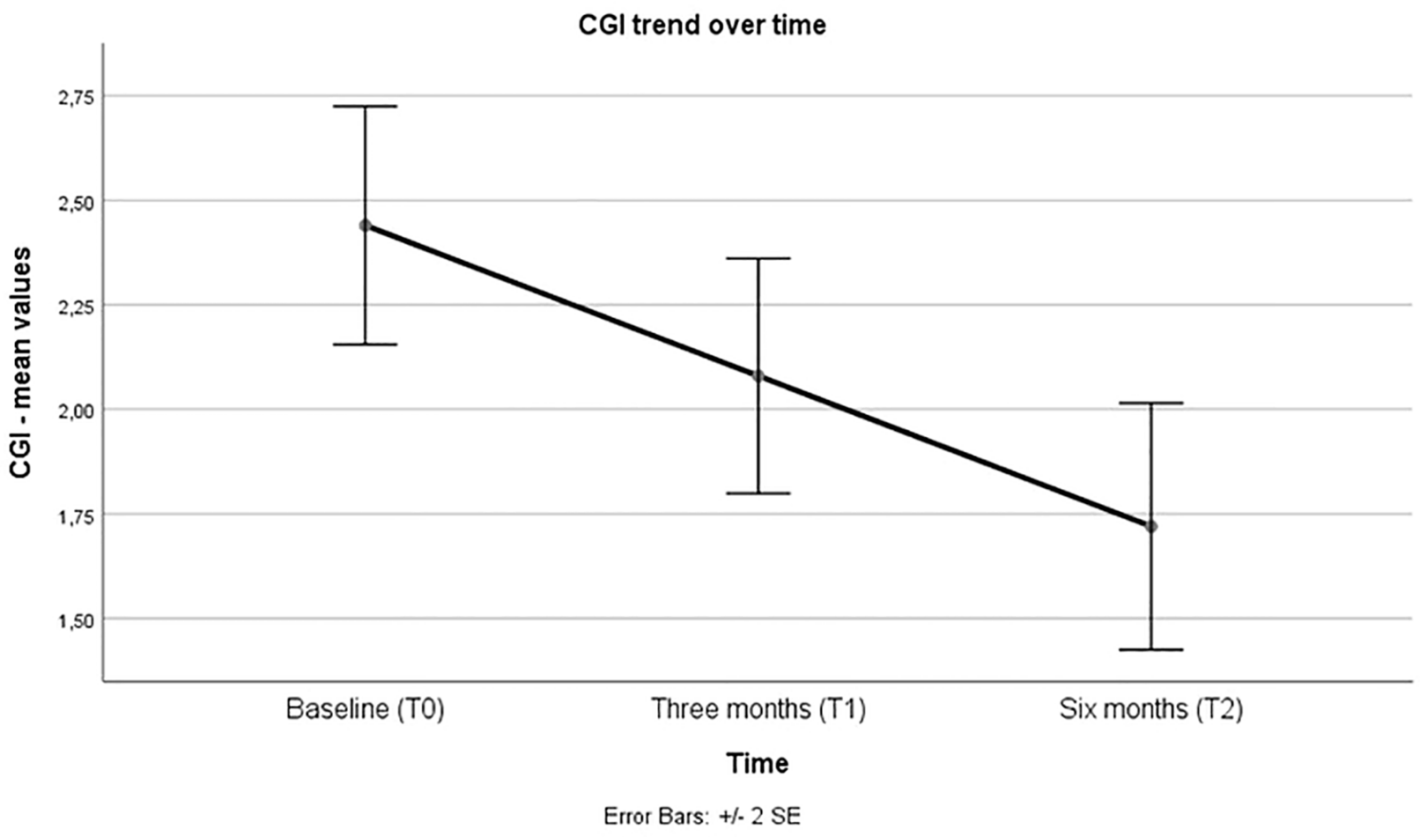
Variation of mean CGI value over observation time.

The values on the CGI scale showed a progressive reduction at the first and second observations compared to baseline, representing a global clinical improvement (**Table 3**; **Figure 3**). This difference was found to be significant in the RM-ANOVA test between T0 and T2 (**Table 4**). These results survive the Bonferroni adjustment.

**Table 3:** CGI test mean values.

CGI - descriptive statistics			
	Mean	Std. dev.	N
CGI - T0	2.44	0.71	25
CGI - T1	2.08	0.70	25
CGI - T2	1.72	0.73	25

**Table 4:** Repeated measurement ANOVA for CGI test.

CGI (T0 - T1 - T2) - RM-ANOVA						
Time		Difference of the mean	Std. error	Sign.^b^	95% CI	
					Inferior limit	Superior limit
T0	T1	0.36	0.17	0.141	-0.08	0.80
	T2	.72^*^	0.13	0.000	0.37	1.06
T1	T0	-0.36	0.17	0.141	-0.80	0.08
	T2	0.36	0.15	0.077	-0.03	0.75
T2	T0	-.72^*^	0.13	0.000	-1.06	-0.37
	T1	-0.36	0.15	0.077	-0.75	0.03

Based on the estimated marginal means.

^*^The difference of the mean is significant for p< 0.05.

^b^Adjustment for multiple comparisons: Bonferroni.

**Figure 4:**
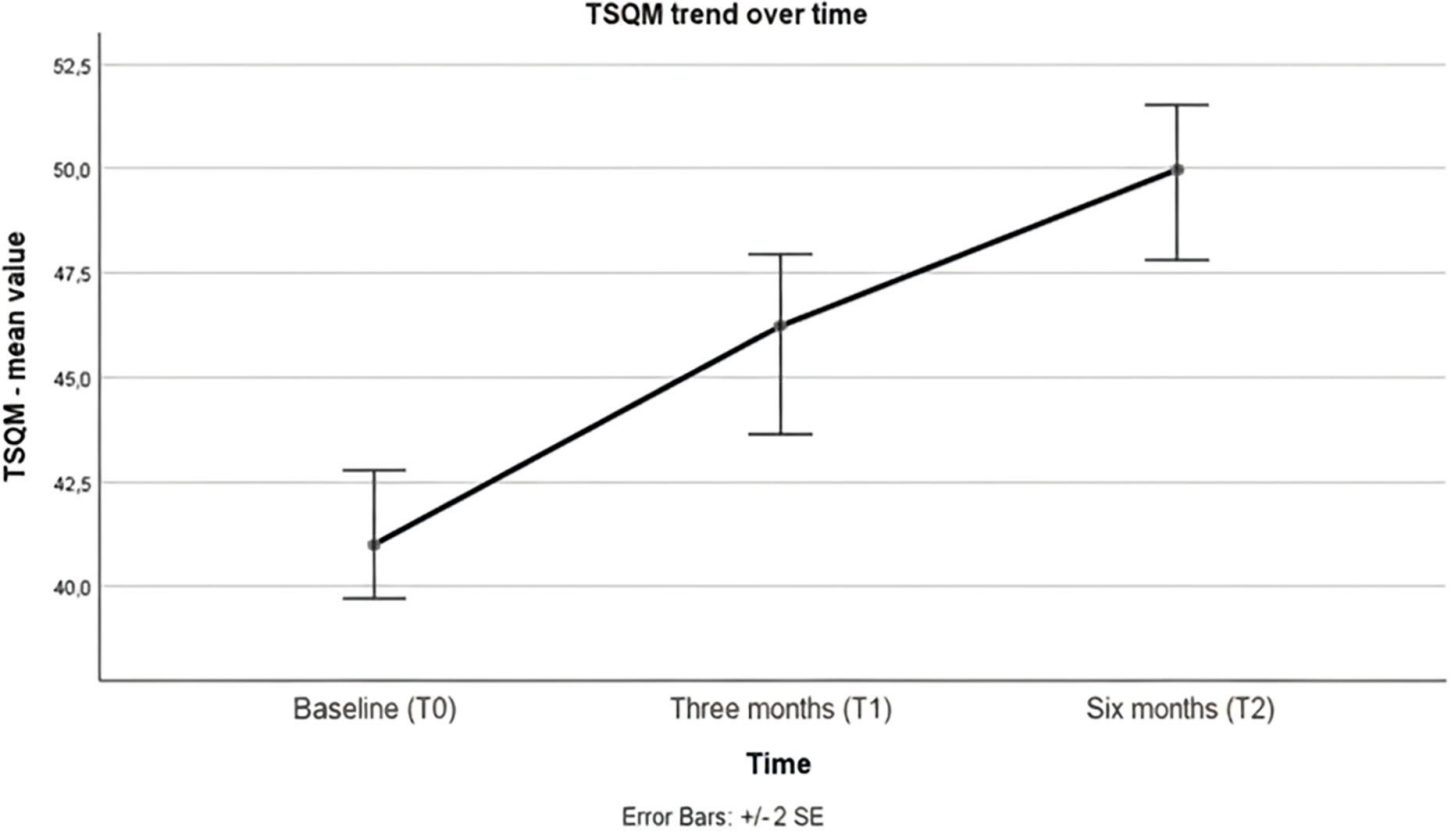
Variation of mean TSQM value over observation time.

The values on the TSQM scale showed a progressive increase at the first and second observations compared to baseline (**Table 5**, **Figure 4**). This difference was found to be significant in the RM-ANOVA test between T0 and T1 and between T0 and T2 (**Table 6**). These results survive the Bonferroni adjustment.

**Table 5:** TSQM test mean values.

TSQM - descriptive statistics			
	Mean	Std. dev.	N
TSQM T0 TOT	41.00	6.26	25
TSQM T1 TOT	46.16	6.52	25
TSQM T2 TOT	49.88	5.54	25

**Table 6:** Repeated measurement ANOVA for TSQM test.

TSQM (T0 - T1 - T2) - RM-ANOVA						
Time		Difference of the mean	Std. error	Sign.^b^	95% CI	
					Inferior limit	Superior limit
T0	T1	-5.16^*^	1.08	0.000	-7.94	-2.37
	T2	-8.88^*^	1.56	0.000	-12.91	-4.84
T1	T0	5.16^*^	1.08	0.000	2.37	7.94
	T2	-3.72^*^	1.41	0.044	-7.35	-0.08
T2	T0	8.88^*^	1.56	0.000	4.84	12.91
	T1	3.72^*^	1.41	0.044	0.08	7.35

Based on the estimated marginal means.

^*^The difference of the mean is significant for p< 0.05.

^b^Adjustment for multiple comparisons: Bonferroni.

The magnitude and direction of change in IAQ, CGI-S, and TSQM 1.4 scores indicated consistent and clinically meaningful improvements over time. Mean differences and corresponding 95% confidence intervals are reported in **Tables 2**, **4**, and **6**. Differences in side effects reported by investigators through specific IAQ test items were considered between baseline and the end of observation. Mean values are reported in **Table 7**. A slight reduction of item mean values has been reported for weight, akathisia and extrapyramidal symptoms, corresponding to an improvement of these effects, while a slight, but insignificant, rise was observed for prolactin and other symptoms. All these changes failed T-test for significancy (**Table 8**).

**Table 7:** Antipsychotic treatment side effects scores reported on IAQ test.

Paired samples statistics				
	Mean	N	Std. dev.	Std. mean error
Weight gain - T0	2.64	25	1.11	0.22
Weight gain - T2	2.48	25	1.08	0.21
Prolactin - T0	0.64	25	1.18	0.23
Prolactin - T2	0.68	25	1.24	0.25
Akathisia - T0	0.96	25	1.13	0.22
Akathisia - T2	0.84	25	1.06	0.21
EPS - T0	0.80	25	1.15	0.23
EPS - T2	0.72	25	1.10	0.22
Other - T0	0.32	25	0.90	0.18
Other - T2	0.44	25	1.04	0.20

T0: baseline, prior to AOM start. T2: six month control.

**Table 8:** Side effect pre-post treatment T-Test. Statistical significance for p<0.05.

Paired samples test								
Side effect baseline vs six months	Paired differences					t	gl	Sign. (two tails)
	Mean	Std. dev	Mean Std. error	95% CI				
				Inferior	Superior			
Weight gain	0.16	0.55	0.11	-0.06	0.38	1.445	24	0.161
Prolactin	-0.04	0.20	0.04	-0.12	0.04	-1.000	24	0.327
Akathisia	0.12	0.52	0.10	-0.09	0.33	1.141	24	0.265
EPS	0.08	0.49	0.09	-0.12	0.28	0.811	24	0.425
Others	-0.12	0.66	0.13	-0.39	0.15	-0.901	24	0.376

Considering self-reported side effects through TSQM, only five patients (18%) complained of the presence of collateral effects, with a perceived medium level of impact of AOM on their quality of life in most cases (80%) and only in a single case the collateral effects were barely tolerated. No treatment discontinuations occurred due to side effects, confirming the overall favorable tolerability profile of AOM in this sample.

Profile of efficacy was evaluated considering the first three items of IAQ test (Positive symptoms; negative symptoms; other efficacy symptoms) confronting baseline (T0) and the end of observation (T2). Results are reported in **Table 9**. All three domains registered a slight decrease, but only the “Negative symptoms” domain variation was significant (T-test: p=0.032) (**Table 10**).

**Table 9:** Pre-post psychopathological domains severity reported on IAQ test.

Efficacy domains statistics				
IAQ domain	Mean	N	Std. deviation	Mean Std. error
IAQ T0 - Positive symptoms	2.04	25	.79	.15
IAQ T2 - Positive symptoms	1.80	25	.81	.16
IAQ T0 - Negative symptoms	1.88	25	1.16	.23
IAQ T2 - Negative symptoms	1.60	25	.91	.18
IAQ T0 - Other efficacy symptoms	.48	25	1.00	.20
IAQ T2 - Other efficacy symptoms	.44	25	.91	.18

T0: baseline. T2: six months.

**Table 10:** T-test on pre-post psychopathological domains severity reported on IAQ test.

Paired samples test								
	Paired differences					t	gl	Sign. (two tail)
	Mean	Std. deviation	Mean std. error	CI 95%				
				Inferior	Superior			
Positive symptoms: T0 – T2	.24	.66	.13	-.03	.51	1.809	24	.083
Negative symptoms: T0 – T2	.28	.61	.12	.02	.53	2.281	24	.032
Other efficacy symptoms: T0 – T2	.04	.35	.07	-.10	.18	.569	24	.574

T0: baseline. T2: six months. Statistical significance for p<0.05.

## Discussion

The primary outcome of this study was the observation of a favorable profile of the efficacy, safety, and tolerability of AOM therapy compared to SOC oral atypical antipsychotic therapy in patients diagnosed/living with schizophrenia. Regarding the psychopathological domains of schizophrenia, the efficacy on positive symptoms was equivalent, while a slight but significant clinical improvement in negative symptoms was observed after the introduction of AOM. From this perspective, AOM resulted in maintained antipsychotic effects, supporting the recovery of affective functions and overall functioning in treated patients.

The global clinical evaluation performed using the IAQ scale shows a gradual and overall improvement in the psychopathological domains of the patients under examination, achieving statistical significance at six months. This observation supports the hypothesis that a prolonged period is necessary for the benefits of injectable treatment to manifest, although they begin to appear in the early months. However, while the beneficial effects associated with LAI therapy were observed, no significant clinical differences were found in the manifestation of known adverse effects of partial agonists. Specifically, there were no statistically significant changes in body weight, the manifestation of EPS, akathisia, or prolactin levels after six months of AOM therapy.

The global clinical assessment performed by the clinician using the CGI-S scale demonstrated a progressive improvement, which was significant after six months of therapy. This evaluation compared the baseline clinical state with SOC oral therapy to the state after the introduction of AOM therapy. Over time, patients receiving the LAI tended to appear less ill, more cognitively and mentally functional, and less burdened by the disease. This aspect is crucial in the clinical management of a patient with schizophrenia, aiming to improve health related quality of life while allowing better treatment adherence, an improved therapeutic relationship, and reduced relapse rates.

Investigating the level of satisfaction with LAI therapy evaluated using the TSQM scale, patients showed progressively greater satisfaction, significantly from the first three-month follow-up, a result confirmed and strengthened at the six-month follow-up. In line with the observed improvements in efficacy and functional impairment, which tended to improve over time, the level of satisfaction with long-acting therapy appears to grow progressively. This result is crucial, as greater satisfaction reduces the likelihood of spontaneous therapy discontinuation and increases adherence. Across the entire sample, only five subjects reported adverse effects, both physical and cognitive, in four cases with a moderate impact on satisfaction and therapy compliance, while only one patient showed poor tolerance to the treatment (asthenia, cognitive slowing), yet treatment adherence of these patients was maintained without discontinuation for the entire observation time. These adverse effects represent a small percentage of the sample, confirming the good tolerability profile of AOM. These side effects, not confirmed through clinical evaluation by a physician, should be considered generic (only described as “physical side effect” of “cognitive side effect”) and self-reported.

Overall, these results demonstrate an advantageous profile of AOM compared to SOC oral therapy. A low incidence of adverse events was observed, combined with efficacy comparable to oral therapy but with greater satisfaction and overall clinical and functional improvement. These results are consistent with findings from real-world studies reporting improvements in patient-reported satisfaction (24) and enhanced global functioning over time (25). Taken together, these findings support the overall efficacy and tolerability profile of AOM compared to oral antipsychotic therapy (10, 17, 18).

Despite the statistical significance and clinical relevance of the observed improvements, it is important to acknowledge that the small sample size and the open-label, single-arm design without a control group limit the generalizability of the findings and may have not considered nonspecific effects related to increased clinical attention or patient expectations. A significant limitation of this study is the number of patients who withdrew, reducing its statistical power. Furthermore, longer-term observation beyond six months could help to define the duration of AOM’s protective effects in limiting relapses, reducing acute psychiatric service use, and improving health related quality of life by reducing the incidence of adverse effects. Another limitation is the absence of an *a priori* sample size calculation, which is inherent to its non-interventional design and may have affected the statistical power to detect small effect sizes.

## Conclusions

This study demonstrates that AOM offers a favorable profile of efficacy, safety, and tolerability in the maintenance treatment of schizophrenia, showing comparable effectiveness on positive symptoms and a significant improvement in negative symptoms compared to SOC oral antipsychotic therapy. The gradual improvement in psychopathological domains underscores the potential benefits of long-term injectable therapy. Patient satisfaction with AOM was progressively increased, indicating a growing acceptance and adherence to the treatment regimen. The low incidence of adverse events further supports the good tolerability of AOM. These findings suggest that AOM could improve health related quality of life and clinical outcomes in patients with schizophrenia, highlighting the need for further long-term studies to confirm these benefits and assess the sustained impact on relapse rates and functional recovery. These findings suggest that AOM could improve health-related quality of life and clinical outcomes in patients with schizophrenia, highlighting the need for further long-term studies to confirm these benefits and assess the sustained impact on relapse rates and functional recovery. Future research should include larger, long-term randomized controlled trials and a deeper evaluation of factors influencing illness trajectories, such as previous treatments, comorbidities, and reasons for treatment discontinuation, to better understand individual variability in response to AOM.

## Data Availability

The raw data supporting the conclusions of this article will be made available by the authors, without undue reservation.
